# The perception of emotion in music by people with hearing loss and people with cochlear implants

**DOI:** 10.1098/rstb.2023.0258

**Published:** 2024-08-26

**Authors:** Brian C. J. Moore

**Affiliations:** Cambridge Hearing Group, Department of Psychology, University of Cambridge, Downing Street, Cambridge CB2 3EB, UK

**Keywords:** music, emotion perception, hearing loss, hearing aids, cochlear implants

## Abstract

Music is an important part of life for many people. It can evoke a wide range of emotions, including sadness, happiness, anger, tension, relief and excitement. People with hearing loss and people with cochlear implants have reduced abilities to discriminate some of the features of musical sounds that may be involved in evoking emotions. This paper reviews these changes in perceptual abilities and describes how they affect the perception of emotion in music. For people with acquired partial hearing loss, it appears that the perception of emotion in music is almost normal, whereas congenital partial hearing loss is associated with impaired perception of music emotion. For people with cochlear implants, the ability to discriminate changes in fundamental frequency (associated with perceived pitch) is much worse than normal and musical harmony is hardly perceived. As a result, people with cochlear implants appear to judge emotion in music primarily using tempo and rhythm cues, and this limits the range of emotions that can be judged.

This article is part of the theme issue ‘Sensing and feeling: an integrative approach to sensory processing and emotional experience’.

## 1. Introduction

In almost all societies, including isolated ones living in remote areas [1], music plays an important role. It enhances social interactions [2], gives pleasure [3], and can convey a wide range of emotions, including sadness, happiness, anger, tension, relief and excitement [4]. It seems likely that links between features of music and emotions are partly culturally based and depend on the form of music in a specific culture, such as the tonal scale used [1,2,5]. For example, in Western music, the major mode (e.g. the notes C, E, G) is associated with happy music while the minor mode (e.g. the notes C, Eb, G) is associated with sad music, but this association is not found for children aged below about 4 years [6]. However, there are probably also features of music that influence emotion regardless of culture [7,8]. For example, a fast tempo is often associated with happy music while a slow tempo is associated with sad music, and dynamic variations may convey excitement or expressiveness, whereas a lack of dynamic variations conveys calmness [9]. For a study of the acoustic features that influence the perception of emotion in music, see Coutinho & Dibben [10].

People with normal hearing usually perceive emotions in music easily and without effort. However, a substantial proportion of the adult population, about 20%, has some degree of hearing loss, and hearing loss becomes increasingly prevalent with increasing age [11]. The most widely used measure of hearing is the pure-tone audiogram, which assesses the lowest sound level at which sinusoids of different frequencies can be detected [12]. The detection thresholds are expressed relative to the average values for young people with no known hearing problems and have units Hearing Threshold Level in decibels (HTL, dB). If the detection threshold at a specific frequency for an individual is, for example, 50 dB HL, this means that the detection threshold is 50 dB higher than ‘normal’, and is referred to as a 50 dB hearing loss. The loss of sensitivity as measured using the audiogram can be alleviated to some extent using hearing aids, which amplify sounds. However, hearing loss is associated with a reduced ability to discriminate the acoustic features in music that may be important for emotion perception and hearing aids may be of limited benefit in alleviating this problem [13].

About 1% of the adult population has hearing loss that is sufficiently severe that they get little benefit from hearing aids, in which case a cochlear implant (CI) in one or both ears may be provided [14]. CIs may also be provided to children with severe or profound hearing loss. Although CIs have been remarkably successful in improving the ability to understand speech for the majority of recipients [15], they do not effectively convey some of the acoustic features that are important for the perception of music. In particular, fundamental frequency (F0), which is associated with perceived pitch, is poorly perceived by people with CIs. This may strongly limit the perception of emotion in music. Nevertheless, many people with CIs report that they like to listen to and perform music [16–18].

This paper reviews the perceptual abilities of people with hearing loss, with and without hearing aids, and of people with CIs, and reviews studies of the perception of emotion in music by such people. Of course, the perception of emotion in speech is also important. This is beyond the scope of the present paper (see [19] for a review).

## 2. Effects of hearing loss on perceptual abilities

### (a) The functioning of the normal ear

**Figure 1.**
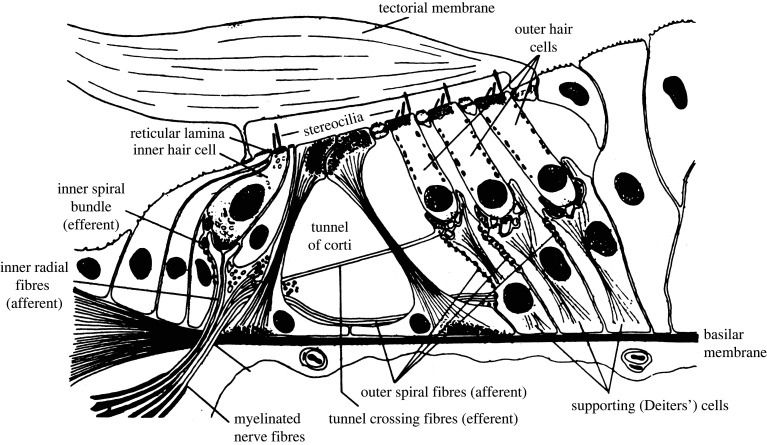
Cross-section of the cochlea showing the basilar membrane and the inner and outer hair cells. From Moore [12]. Published by permission of the author.

Figure 1 shows a cross-section of the cochlea, which is the snail-shaped part of the inner ear. Sound evokes a wave on the basilar membrane (BM) which travels from the base towards the apex. In response to a sinusoidal input (sometimes called a ‘pure tone’), the wave builds up and then dies away, and the position of peak vibration varies systematically with the input frequency; low-frequency sounds produce peak vibration near the apex and high-frequency sounds produce peak vibration near the base, with a continuous gradation in between. When a complex sound with multiple sinusoidal frequency components, such as a note produced by a musical instrument, is presented, each component leads to a distinct peak in the vibration pattern on the BM, provided that the components are sufficiently separated in frequency. This forms the basis for frequency selectivity, the ability to ‘hear out’ the individual frequency components in a complex sound [20–22]. The vibration pattern around the peak in response to a sinusoidal component is amplified and sharpened by an active mechanism that depends on the motor activity of the outer hair cells (OHCs) [23]; these form 3–5 rows running along the length of the BM. The vibration is detected via the inner hair cells (IHCs), which form a single row running along the length of the BM. Electrical currents flowing through the IHCs lead to a release of neurotransmitter at the synapses that connect the IHCs to the nerve fibres that make up the auditory nerve; about 12–15 nerve fibres have synapses with each IHC [24]. The release of neurotransmitter at the synapses results in action potentials (spikes) in the auditory nerve that convey information about sound from the cochlea to the brain. The number of spikes evoked at a given place on the BM increases with increasing amount of vibration at that place.

Information about the frequencies of sounds is carried in the auditory nerve in two forms. The first is related to the distribution of vibration along the BM; the position of each peak in the vibration pattern conveys information about the frequency of the sinusoidal component that gave rise to that peak. This is often referred to as ‘place’ coding [12]. The second form of information is connected with the detailed time pattern of the nerve spikes. The nerve spikes evoked by vibration at a given place on the BM are synchronized to a specific phase of the waveform at that place, a property known as ‘phase locking’ [25,26]. As a result, the intervals between successive nerve spikes are clustered around integer multiples of the period of the sound that is driving the response at that place. For example, in response to a 1000 Hz sinusoid, the intervals between nerve spikes cluster around intervals of 1, 2, 3, 4, 5 … ms. Phase locking is precise at low and medium frequencies, but becomes weaker at high frequencies [26]. The upper-frequency limit varies across species and the limit in humans is not known [27] but may be as high as 8–10 kHz [28,29].

The detailed temporal pattern of nerve spikes evoked by sinusoids and by complex sounds is referred to in this paper as ‘temporal fine structure (TFS)’. It is known that TFS plays a role in the localisation of sounds with frequencies up to about 1400 Hz [30], but its role in other aspects of perception is still debated [27,31]. There is at least some evidence that TFS information is used for discriminating changes in the frequency of sine waves [28,29,32], for discriminating the fundamental frequency of complex sounds [33], and for discriminating sounds with the same envelope repetition rate but different TFS [34–36].

Hearing loss can be associated with dysfunction of the OHCs, the IHCs, the synapses between the IHCs and neurons, and the neurons themselves, and any combination of these [37–40]. The perceptual consequences of each form of dysfunction are described next.

### (b) Outer hair cell dysfunction

Dysfunction of the OHCs is common following noise exposure or with increasing age [37,40,41]. This dysfunction impairs the operation of the active mechanism, and this has three main consequences. Firstly, it reduces the amount of BM vibration around the peak of the vibration pattern, especially for low-level sounds. The perceptual correlate of this is an elevation in the absolute threshold—the lowest detectable sound level, which is what is measured by the pure-tone audiogram. The maximum amplification produced by the active mechanism is about 55 dB [42], so the maximum hearing loss that can be produced by OHC damage alone is also about 55 dB.

A second consequence of OHC damage is reduced frequency selectivity. Each point on the BM behaves like a bandpass filter whose centre frequency (CF) varies with position along the BM. These filters, often called the auditory filters, have bandwidths at medium and high CFs that are 12–13% of the CF, for people with normal hearing [43]. Dysfunction of the OHCs causes the filters to broaden by a factor of up to 4 [38,44,45]. This reduces the ability to determine the spectral shape of sounds, which is important for distinguishing spoken or sung speech sounds and for distinguishing different musical instruments. It also reduces the ability to hear out individual frequency components in a complex sound [46] and to hear out individual instruments or groups of instruments in a mixture, which is a problem experienced by people with hearing loss [47]. Finally, it may reduce the clarity of the pitch of complex sounds because of the poorer resolution of the low harmonics [48].

A third consequence of OHC dysfunction is an effect called loudness recruitment [49,50]. Assume that the level of a sound is gradually increased from a very low starting value. For a person with hearing loss, the level at which the sound first becomes detectable is higher than for a person with normal hearing. However, once the sound level exceeds the detection threshold, the loudness grows more rapidly than normal. At high sound levels, the loudness in an ear with OHC dysfunction ‘catches up’ with the loudness in a normal ear. As a result, a person with OHC dysfunction can only hear comfortably over a small range of sound levels; this is described as having a reduced dynamic range. Loudness recruitment occurs because the OHCs in a normal ear amplify the response of the BM to weak sounds but the amplification progressively reduces as the sound level increases. Essentially, normal ears have a form of amplitude compression or automatic gain control. In an ear with OHC dysfunction, this compression is reduced or lost altogether.

For a person with loudness recruitment, fluctuations in sound level appear exaggerated. For example, an amplitude-modulated sound appears to fluctuate more when presented to an ear with loudness recruitment than when presented to a normal ear [51]. When listening to music, the dynamic aspects may appear exaggerated.

### (c) Inner hair cell, synaptic and neural dysfunction

IHC, synaptic and neural dysfunction all reduce the number of nerve spikes evoked by a sound in the auditory nerve. Synaptic dysfunction, called synaptopathy, is associated with both noise exposure [40,52] and increasing age [24]. Unless the dysfunction is extreme, this has little effect on the threshold for detecting sounds, since only a few nerve spikes are sufficient for a sound to be detected [53]. This type of dysfunction has been called ‘hidden hearing loss’ [54,55] or ‘hidden hearing disorder’ [56], because its effects are not apparent in the pure-tone audiogram. The reduced number of spikes leads to less precise neural coding of the properties of sounds. It is somewhat controversial how much IHC, synaptic and neural dysfunction is required before its perceptual effects become manifest [57]. However, it seems likely that IHC, synaptic and neural dysfunction cause poorer discrimination of sounds, and may contribute to reduced sensitivity to the TFS of sounds [58] and to poor pitch perception [59] and sound localization [60]. IHC, synaptic and neural dysfunction may also adversely affect the ability to discriminate changes in the envelopes of sounds [61].

When the IHC, synaptic or neural dysfunction is nearly complete over a certain region along the BM, very few or no spikes are generated from that region, so little or no information about BM vibration is conveyed in the auditory nerve. Such a region is referred to as a ‘dead region’ [62,63]. When the hearing loss at a given frequency is 70 dB HL or more, there is more than a 50% chance of there being a dead region at the place on the BM tuned to that frequency [64,65]. Hence, dead regions are common among those with severe or profound hearing loss. Tones producing peak BM vibration within a dead region are often perceived as highly distorted or noise-like [66], and often do not have a clear pitch [67].

## 3. Benefits and limitations of hearing aids

### (a) Compensation for threshold elevation and loudness recruitment

Most hearing aids incorporate signal processing to compensate to some extent for threshold elevation and loudness recruitment. Usually, this is done by filtering the sound into several frequency bands or ‘channels’ and applying independent automatic gain control (AGC) to the signal in each channel [68]. With AGC, weak sounds are strongly amplified so as to restore their audibility, but the amplification is progressively reduced as the sound level increases, so that intense sounds are not amplified at all, or are even attenuated. This partially compensates for the effects of loudness recruitment. The amplitude compression that occurs in a normally functioning cochlea is very fast acting [69], i.e. the amplification changes rapidly with changes in the input sound level. Hence, it might be expected that the AGC in hearing aids would be most effective if it also was fast acting. However, despite many research studies, there is no clear consensus as to whether fast or slow compression is preferable [70,71]. Based on the subjective judgements of hearing-impaired people, it appears that slow compression is slightly preferred over fast compression for listening to music [72].

It should be noted that multi-channel compression does not compensate fully for the effects of threshold elevation and loudness recruitment. The amplification of weak sounds is usually not sufficient to fully restore their audibility, and slow-acting AGC does not compensate adequately for rapid fluctuations in level, such as can occur in music. Hence, dynamic changes in music can appear exaggerated, and users of hearing aids still complain that some sounds are too loud [73]. The altered perception of dynamic changes may adversely affect the perception of emotion in music.

### (b) Compensation for loss of frequency selectivity

Hearing aids at present do not incorporate any direct form of compensation for the effects of reduced frequency selectivity. Some experimental signal-processing schemes have been evaluated that process the short-term spectra of sounds so as to increase the contrast between peaks and valleys [74–76] or so as to enhance spectral changes over time [77], but these have shown only modest benefits, and they have not been implemented in commercial devices. Hearing aids can compensate to some extent for a reduced ability to understand speech in noise via the use of directional microphones [78,79] and via the use of deep neural networks [80], but such systems are of limited benefit when listening to music, especially when trying to ‘hear out’ the individual instruments.

### (c) Compensation for the effects of inner hair cell, synaptic and neural dysfunction

Hearing aids at present do not incorporate any direct form of compensation for the effects of IHC, synaptic and neural dysfunction.

## 4. Perception of sound via cochlear implants

As described in the introduction, about 1% of the adult population has hearing loss that is sufficiently severe that they get little benefit from hearing aids. In a large proportion of these people, the disorder is in the cochlea rather than in the central nervous system, and the auditory nerve is partially intact. In such people, it is possible to create a sensation of sound by electrical stimulation of the auditory nerve. This is done using CIs. CIs are often also provided for children with severe or profound hearing loss. A CI has the following components: an external part, usually positioned behind the ear, that includes microphones for picking up the sound, and a signal processor; a coil positioned on the surface of the scalp that receives signals from the processor and transmits them across the skin; and a coil implanted under the skin that receives the transmitted signal, ‘decodes’ it, and sends appropriate signals to individual electrodes of an array inserted into the cochlea.

The sound processor usually includes an AGC system, followed by an array of bandpass filters to split the signal into ‘channels’. The envelopes of the channel signals are extracted, subjected to amplitude compression, and used to control the amplitude or duration of biphasic electrical pulses that are delivered to the implanted electrodes. The envelope magnitude in each channel is typically coded by pulse magnitude (current) or pulse duration. Increases in either of these quantities lead to increased neural spike rates in the auditory nerve and hence to increasing loudness. With electrical stimulation, the rate of increase of spike rate with increasing current (or pulse duration) is very rapid [81,82]. As a result, small changes in current or pulse width lead to large changes in loudness. This is the reason why most CIs include an initial AGC system and instantaneous amplitude compression of the channel envelopes. However, with these systems, dynamic changes in a piece of music may be poorly conveyed [83].

The use of an array of electrodes makes it possible selectively to stimulate groups of neurons within the cochlea. The outputs of channels tuned to high frequencies are delivered to the base of the cochlea, giving a sensation described as ‘sharp’, while the outputs of channels tuned to low frequencies are delivered closer to the apex, giving a ‘dull’ sensation [84]. This crudely simulates place coding in the auditory system. Unfortunately, the electrical current produced by stimulation of a given electrode spreads considerably along the cochlea. This limits the effective number of separate ‘channels’ for electrical stimulation. Effectively, the frequency selectivity of CI users is poor, primarily because of current spread.

Most CI systems do not convey information about the TFS of the channel signals, except in some systems for the channels tuned to the lowest frequencies. This is because CI users seem to be relatively insensitive to TFS, except for very low frequencies [85]. As a result, most CI users do not experience a clear sensation of pitch, especially for mixtures of the sounds of different instruments [16].

In summary, CIs lead to unnatural perception of dynamic changes in sound level, poor frequency selectivity due to current spread, and poor pitch perception due to the combined effects of current spread and limited or no TFS coding. All of these effects may adversely affect the perception of emotion in music. CI users are able to discriminate rhythms as well as people with normal hearing [86]. However, CI users perform markedly more poorly than people with normal hearing in judging melodic contours [87]. They are also poor at discriminating differences in musical harmony and in judging consonance or dissonance [88] (for a review, see [16]). Despite their limited ability to discriminate some features of music, many CI users report that they enjoy listening to music, and some play musical instruments.

## 5. Perception of emotion in music for people with hearing loss

There are few studies of the perception of emotion in music by people with hearing loss, with or without hearing aids. One study [89] investigated whether students with hearing loss assigned the same emotions to music as students without hearing loss. One group of participants was composed of 31 students at a state school for the deaf in the USA who had hearing loss from early in life and used American Sign Language. They had hearing losses ranging from moderate to severe. A control group of 31 normal-hearing (NH) participants was recruited from students at neighbouring elementary and junior high schools. For both groups the ages of the participants ranged from 6 to 14 years with a mean of 10 years, and roughly half in each group were female.

Twelve film score excerpts, composed to depict the emotions of happiness, sadness and fear, were used as the musical stimuli. Each excerpt lasted 15 s and was presented via stereo loudspeakers set on tables. The hearing-impaired participants were tested using their usual hearing aids. For them, the sound level of each excerpt was set at the highest level not resulting in distortion of the music. The hearing-impaired participants were encouraged to sit at a distance from the loudspeakers that resulted in their most comfortable listening level. The NH participants were tested at ‘normal classroom listening level’. Participants were asked to assign an emotion after listening to each excerpt, choosing one of happiness, sadness and fear. Participants aged 6–8 years used response sheets that had a face drawing depicting the emotion paired with the word. Older participants used response sheets that had only the emotion words.

The responses of the NH participants were significantly more in agreement with the composers' intent than the responses of the hearing-impaired participants. There was no significant effect of age or gender. Analyses of the relationship between the physical characteristics of the excerpts and the responses of the hearing-impaired participants suggested that spectral shape and rhythm were the most effective musical features in transmitting emotion. Generally, the performance of the hearing-impaired participants was better for excerpts that had a single clear melodic line, such as a flute solo, consistent with their expected difficulty in hearing out the individual instruments in a complex piece of music with many instruments playing.

Another study [90] compared three groups of students:

(1) 30 students with bilateral congenital moderate-to-severe hearing loss who were studying in a school for the hearing impaired. Their mean age was 17 years (range 15–19 years).
(2) 30 students with acquired hearing loss of a similar degree to that of group 1, who were attending an audiology clinic in a university hospital centre. Their mean age was 22 years (range 20–25 years).
(3) A control group of 30 age- and gender-matched NH students. Their mean age was 20 years (range 16–28 years). Students in groups 1 and 2 usually wore bilaterally fitted hearing aids, but these were not worn during testing.

The musical stimuli were three sequences of music intended to convey the emotions of sadness, happiness and fear, each with a duration of 60 s. They were presented at the most comfortable listening level, which was selected individually for each student. After a given sequence had been presented, the student was shown three lists of words, one related to sadness, one to happiness, and one to fear. The student was asked to point to the list of words that best matched their emotions. Emotion recognition was significantly poorer for those with congenital hearing loss than for the other two groups. Emotion recognition did not differ significantly for groups 2 and 3. There were no significant effects of age or gender. Note that the lack of difference between groups 2 and 3 contrasts with the worse perception of emotion in speech found for people with acquired moderate-to-severe hearing loss [19].

The reasons for the poorer performance of the students with congenital hearing loss are not clear. However, it seems likely that exposure to music early in life with normal or near-normal hearing, as occurred for groups 2 and 3, helps the individual to learn the characteristics of music that convey emotion, and once those characteristics are learned, they are sufficiently robust that they can still be perceived even when the individual acquires a hearing loss. Even though the individuals with congenital hearing loss had been fitted with hearing aids, they still would have had deficits in auditory processing from an early age, and this may have limited their ability to learn the acoustical features in music that convey emotion.

Of the few studies examining the perception of emotion in music by hearing-impaired adults, some have appeared only as conference abstracts, so details are sparse. In one study [91], three groups were tested: (1) hearing-impaired people who did not use hearing aids; (2) hearing-impaired hearing-aid users, tested while wearing their usual hearing aids; and (3) NH controls. Participants were asked to choose one of five emotions (anger, fear, happiness, sadness and tenderness) after listening to each of 50 15-s musical excerpts that had been shown to reliably convey one of the five emotions for NH participants [92]. The NH participants performed better than the two groups of hearing-impaired participants, but performance did not differ significantly for the unaided and aided hearing-impaired listeners. The lack of effect of hearing aids is consistent with the results of studies of the perception of emotion in speech [19].

In another study [93], three groups of participants, all aged 60 years or more, were tested: (1) 18 hearing-impaired individuals who did not use hearing aids; (2) 18 hearing-impaired hearing-aid users, tested while wearing their usual hearing aids; and (3) 18 NH controls. Arousal ratings and skin conductance measures were obtained after each participant listened to each of 24 excerpts of film music that were previously validated as conveying one of four emotions: happy, sad, fearful and tender. These four emotions were chosen because they represent a crossing of arousal (high and low) and valence (positive and negative). For each group, an ‘arousal range’ was calculated as the average arousal rating of happy and fearful excerpts minus the average arousal rating of tender and sad excerpts. A similar scheme was used to determine a ‘skin conductance range’. For music with a negative valence (sad, fearful), neither arousal nor skin conductance differed significantly across groups. For music with a positive valence, both the arousal range and the skin conductance range were smaller for the hearing-impaired individuals who did not use hearing aids than for the other two groups.

The results of these two studies with adults are not entirely consistent. Both studies suggest that hearing loss has an adverse effect on the perception of emotion in music, but the study of Russo and Fanelli [91] suggests that hearing aids were not effective in compensating for this adverse effect while the study of Russo & Scholey [93] suggests that hearing aids were effective, at least in terms of compensating for changes in the extent of the arousal evoked by the music.

Overall, the results suggest that moderate-to-severe hearing loss adversely affects the perception of emotion in music, and the adverse effects are greater for individuals with congenital hearing loss than for individuals with acquired hearing loss. Hearing aids are of some help in alleviating the adverse effects but are not fully effective. Individuals with hearing loss perceive emotion better for pieces of music with a single clear melodic line than for more complex pieces, perhaps because a single clear melodic line leads to a clearer perception of pitch. Generally, it is likely that hearing-impaired individuals tend to make more use of relatively robust cues for emotion, like dynamic changes in level and tempo. Given the somewhat conflicting results of the limited published work in this area, more research is clearly needed.

## 6. Perception of emotion in music for people with cochlear implants

As described in §4, CIs lead to unnatural perception of dynamic changes in sound level, poor frequency selectivity due to current spread, and poor pitch perception due to the combined effects of current spread and limited or no TFS coding. In addition, CI users have a very limited ability to hear out the individual instruments or voices in a piece of music and to judge musical harmony [16,85], so their ability to judge emotion in music using pitch or harmony is likely to be markedly worse than normal. Despite this, many people with CIs regularly listen to or participate in music, and report that this is enjoyable and evokes emotions [16,17]. While there are many studies of the perception of emotion by users of CIs, those studies have mostly been restricted to judgements of happy versus sad, perhaps reflecting the limited range of emotions that can be conveyed effectively via CIs. Many studies have assessed the effect of tempo, usually specified as the number of beats per minute (bpm). Recently, evidence has been presented that the critical temporal feature is the mean onset-to-onset time of successive notes, rather than the tempo in bpm [94]. However, for simplicity, the term tempo will be used here, since that was the quantity specified in most studies. In what follows, studies using children are described before studies using adults.

Hopyan *et al.* [95] assessed the ability to identify sadness or happiness in music for 14 prelingually deaf children (aged 7–13 years) who had received unilateral CIs at an early age (mean age at CI activation of 2.9 years) and 18 age- and gender-matched NH controls. The stimuli were 32 brief excerpts of piano music taken from the classical repertoire of Western music, presented via a loudspeaker (the sound level was not specified). The happy-sounding excerpts were in the major mode and had a rapid tempo, while the sad-sounding excerpts were in the minor mode and had a slow tempo. After each excerpt had been presented, the child was shown two cartoon faces, one happy and one sad, and was asked to point to the face that matched the emotion in the music. The NH group performed near ceiling (mean score of 97% correct). The CI group performed more poorly on average (mean score of 77.5% correct, with a range from 53% to 91% correct), but still performed well above chance. For the CI group, there was no significant correlation between emotion identification scores and age at CI activation or time since CI activation.

In a similar study, Volkova *et al.* [96] compared the ability of 14 prelingually deaf children (aged 4–6 years) with bilateral CIs and 18 NH children (also aged 4–6 years) to identify happiness or sadness in music. The stimuli were 10 10-s synthesized piano excerpts, five intended to sound happy and five intended to sound sad. The happy-sounding excerpts were in the major mode and had a rapid tempo (mean of 137 bpm), while the sad-sounding excerpts were in the minor mode and had a slow tempo (mean of 46 bpm). All notes had the same amplitude. The excerpts were replayed from stereo loudspeakers at a level of about 65 dB sound pressure level (SPL). Following the presentation of each excerpt, the child was asked to indicate which of the two cartoons matched the music. One cartoon was of a child laughing and the other was of a child wiping tears from her eyes. The NH children performed close to ceiling (98% correct). On average, the children with CIs performed more poorly than the NH children, but most scored above chance. The scores for the children with CIs ranged from about 50% to 100%. For the CI users, the association between duration of implant use and performance was significant, in contrast to the findings of Hopyan *et al.* [95]. The authors suggested that ‘auditory experience enabled some children to learn which emotional labels are linked to which acoustic cues in speech and music’.

Giannantonio *et al.* [97] assessed how judgements of happiness or sadness in music by children were affected by auditory experience in early development. The participants were 16 NH children (aged 6.5 to 13 years) and 42 children with hearing loss who used bilateral auditory prostheses; 31 had CIs in both ears (age range 7 to 14 years) and 11 had a CI in one ear and a hearing aid in the other ear (age range 7 to 14 years), because they had some acoustic hearing in that ear. The participants were asked to judge whether musical excerpts sounded ‘happy’ or ‘sad’. There were four conditions, each with 32 piano selections. The first condition (Original tunes) contained musical excerpts lasting 8–11 s drawn from the Western classical music repertoire, chosen so that half evoked a sense of happiness and the other half evoked a sense of sadness. In this condition, happy excerpts had the major mode and reasonably fast tempi (80–255 bpm) and sad excerpts had the minor mode with slower tempi (20–100 bpm). In the second ‘Mode-changed’ condition, the mode was changed from major to minor or vice versa. In the third ‘Tempo-changed’ condition, all tempi were set to 80 bpm, slower than for most of the originally happy tunes and faster than for most of the originally sad tunes. In the fourth condition, ‘Mode-changed’ and ‘Tempo-changed’ were combined.

The NH children based their emotions judgements on both the mode and tempo cues. Musical training accentuated the reliance on the mode cue and this reliance increased with increasing age. The children with bilateral CIs relied mainly on tempo cues. The children with some residual acoustic hearing in one ear showed some reliance on mode, but not as much as for the NH group. The authors concluded that, in a Western culture, access to acoustic hearing in early life promotes a preference for mode rather than tempo cues, and this preference is enhanced by musical training. However, the use of mode depends on having some acoustic hearing, and CI users without residual acoustic hearing rely primarily on tempo to judge sadness or happiness, regardless of musical experience.

In a similar study [98], 16 children with a CI in one ear only (and no usable hearing in the other ear) were compared with 16 NH children. Both groups had a mean age of about 13 years. The task and stimuli were similar to those of Giannantonio *et al.* [97]. When the excerpts had both the original mode and tempo, the CI users discriminated between happy and sad music with an accuracy well above chance (87.5%) but significantly below that for the NH children (98%). The CI group primarily used tempo cues, whereas the NH children relied more on mode cues. The CI users had significantly slower response times than the NH children across all conditions, suggesting that the CI users found the task more difficult.

Caldwell *et al.* [99] compared the extent to which adult CI users and NH individuals relied on tempo and mode in judging sadness versus happiness in music. They tested 15 post-lingually deafened CI users (average age about 52 years) and 16 NH controls (average age about 24 years). They used as stimuli four-bar melodies played on a piano with choral accompaniment, each lasting 20 s. They used two tonalities (major versus minor) and two tempi (presto versus largo), combined in all four possible ways. The combination major key/fast tempo was intended to promote ‘happy’ judgements while the combination minor key/slow tempo was expected to promote ‘sad’ judgements. The other two combinations, major key/slow tempo and minor key/fast tempo, were ambiguous. After listening to each melody, the participant was asked to rate the emotion on a scale ranging from −5 (sad) to +5 (happy). The results showed an overall effect of tempo for both groups, scores being higher (indicating greater judged happiness) for the fast tempo. The NH group also gave higher scores for the major than the minor mode, but the CI group did not. The CI group rated stimuli of the same tempo similarly, regardless of changes in mode. These outcomes were unaffected by the degree of musical training of the participants. Overall, these results show that while NH adults from a Western culture use both mode and tempo to judge sadness versus happiness in music, CI users rely mostly on tempo and do not use mode.

Ambert-Dahan *et al.* [100] assessed how adults who had received a CI after post-lingual progressive hearing loss perceived emotions in music. Thirteen CI users with good verbal comprehension and 13 NH participants matched for age, gender and education listened to 40 short musical excerpts intended to express one of the following emotions: fear, happiness, sadness and peacefulness. The excerpts followed the rules of the Western tonal system and were based on a melody played on a synthetic piano with an accompaniment. The excerpts had a regular temporal structure except for a few fearful excerpts. The happy excerpts were written in a major mode at a rapid average tempo, with the melodic line lying in the medium–high range of fundamental frequencies, and the sustain pedal was not used. The sad excerpts were written in a minor mode at a slow average tempo, and the sustain pedal was used. The peaceful excerpts were written in a major mode, had an intermediate tempo, and were played with sustain pedal and arpeggio accompaniment. Most fearful excerpts were regular and consonant, with various tempos that ranged from slow to rapid, and contained minor chords. The excerpts were presented via stereo loudspeakers at 70 dB SPL. The participants were asked to rate how much each excerpt expressed each of the four emotions, and to judge their emotional valence (unpleasant–pleasant) and arousal (relaxing–stimulating). Although the CI users performed above chance, their mean per cent correct scores were generally lower than for the NH participants for happy, scary and sad excerpts, but not for peaceful excerpts. The CI users also showed deficits in perceiving the arousal of musical excerpts, whereas rating of valence were similar to those for the NH group.

Paquette *et al.* [101] assessed perception of the emotions of happiness, sadness, fear and neutrality in music for 11 experienced unilateral CI users aged 21 to 63 years. The stimuli were brief musical phrases (mean duration = 1.8 s) played on the clarinet or violin and presented through stereo loudspeakers at a level of 70 dB SPL. After listening to each excerpt, the participant rated it using continuous horizontal visual analogue scales. Initially, a scale was displayed for each emotion (happiness, sadness, fear and neutrality), and the participant rated how much the excerpt expressed each emotion on a scale going from ‘Absent’ to ‘Present’. The participant then rated their confidence in their emotion ratings, from ‘Not at all confident’ to ‘Extremely confident’. On the second page, the participant rated the emotional valence of the excerpt from ‘Extremely negative’ to ‘Extremely positive’ and its level of arousal from ‘Not at all arousing’ to ‘Extremely arousing’.

Only the happy excerpts were recognized significantly above chance. The sad excerpts were often identified as happy or neutral, while the fear excerpts were often identified as sad or neutral. However, the happy excerpts were generally correctly identified as positive and arousing emotion and the fear excerpts were generally identified as negative, so the CI users had at least some ability to judge emotional valence, as was found by Ambert-Dahan *et al.* [100].

Overall, the results suggest that users of CIs, both children and adults, have some ability to judge some basic emotions in music, such as happy or sad, but they do this based largely on easily perceived aspects of the music, such as tempo, rather than on F0 or harmony. Experience of music early in life may improve the ability of CI users to judge emotion in music, but more research is needed to clarify the role of early experience. CI users have some ability to judge arousal for music, although this ability is poorer than for NH listeners, perhaps partly because CIs lead to an unnatural perception of the dynamic changes in music. However, CI users have a reasonably good ability to judge valence, i.e. to judge whether the evoked emotion was positive or negative.

## 7. Research needs and open issues

The two main features that have been manipulated in studies of the perception of emotion in music by people with hearing difficulties, namely mode and tempo, represent only a sub-set of the features that can affect emotion perception. Studies are needed to explore other relevant features, such as variations in tempo, manipulation of the silent intervals between notes, and spectral changes over time.

An open issue is whether some aspects of the signal processing used in hearing aids and CIs might actually impair the perception of emotion in music, offsetting any benefits arising from improved audibility. Research is needed to explore how signal processing such as multi-channel compression influences the perception of emotion in music.

## 8. Conclusion

Moderate-to-severe hearing loss adversely affects the perception of emotion in music, and the adverse effects are greater for individuals with congenital hearing loss than for individuals with acquired hearing loss. Hearing aids are of some help in alleviating the adverse effects, but are not fully effective. Individuals with hearing loss perceive emotion better for pieces of music with a single clear melodic line than for more complex pieces, perhaps because a single clear melodic line leads to a clearer perception of pitch.

Both children and adult users of CIs have some ability to judge some basic emotions in music, such as happy or sad, but they do this based largely on easily perceived aspects of the music, such as tempo, rather than on F0 or harmony. Experience of music early in life may improve the ability of CI users to judge emotion in music, but more research is needed to clarify the role of early experience. CI users have some ability to judge arousal in music and have a reasonably good ability to judge valence, i.e. to judge whether the evoked emotion was positive or negative.

## Data Availability

This article has no additional data.
